# A qualitative study of the ecology of stigma experiences - An application of the ecological model to stigma experiences of trans-women from Ghana. BSGH019

**DOI:** 10.1371/journal.pgph.0006336

**Published:** 2026-04-24

**Authors:** Osman Wumpini Shamrock, Chris Guure, Jaquetta Marie Reeves, Zhao Ni, DeAnne Turner, Aliyu Haruna, Isaac Gyasi Nimako, Natalie M. Leblanc, Leo Wilton, Gamji Rabiu Abu-Ba’

**Affiliations:** 1 Behavioral, Sexual, and Global Health Lab, School of Nursing, University of Rochester, Rochester, New York, United States of America; 2 Behavioral, Sexual, and Global Health Lab, North Legon, Accra, Ghana; 3 School of Nursing, University of Rochester, Rochester, New York, United States of America; 4 Department of Biostatistics, School of Public Health, University of Ghana, Accra, Ghana; 5 College of Nursing and Health Innovation, The University of Texas at Arlington, Arlington, Texas, United States of America; 6 School of Nursing, Yale University, New Haven, Connecticut, United States of America; 7 College of Nursing, University of South Florida Tampa, Tampa, Florida, United States of America; 8 Center for Interdisciplinary Research on AIDS, School of Public Health, Yale University, New Haven, Connecticut, United States of America; 9 Department of Human Development, State University of New York at Binghamton, Binghamton, New York, United States of America; 10 Faculty of Humanities, University of Johannesburg, Johannesburg, Gauteng province, South Africa; 11 Department of Public Health Sciences, University of Rochester, Rochester, New York, United States of America; Islamic Azad University South Tehran Branch, IRAN, ISLAMIC REPUBLIC OF

## Abstract

Trans-women in Ghana experience stigma from institutions, social groups, and individuals. Yet, studies are needed to provide a comprehensive understanding of the nature of stigma experienced by trans-women, especially in Ghana and West Africa. This study employs an ecological model to qualitatively explore the ecology of stigma experiences of trans-women living in urban slums in Accra, Ghana (N = 20). Findings revealed stigma across various ecological levels. At the public policy level, the LGBTQ+ bill emerged as a key driver of stigma. Various subcategories related to this bill included: (a) Restricted freedoms of expression and isolation from harm, (b) LGBTQ+ bill potential for facilitating violence, and (c) fear of arrest and jail time. At the institutional level, trans-women encountered stigma in three areas. The first was in the healthcare sector involving rude gestures and behaviors. The second was from law enforcement officers and included (a) harassing attitudes from law officers, and (b) extortion. Community-level stigma encompassed: (a) insults, name-calling, (b) possible harm from gender expression, and (c) fear of attacks. At the interpersonal level, Trans-women experienced: (a) invasion of privacy, (b) leaving home due to safety, (c) threat of bodily harm, and (d) rejection. At the individual/self-stigma level, trans-women experienced (a) self-doubt and internal struggles, and (b) sense of regret and guilt. Findings highlight the unique experiences and pervasive and multi-layered stigma faced by trans-women across different levels of society. Addressing stigma experiences requires a multi-tiered approach that tackles issues from public policy to individual self-acceptance.

## Introduction

According to Pescosolido et al. [1] “stigma complex as the set of interrelated, heterogeneous system structures, from the individual to the society, and processes, from the molecular to the geographic and historical, that constructs, labels, and translates difference into marks” (P.101). Stigma is a significant problem for non-heterosexual, gender diverse people and individuals who identify with the LGBTQ+ community [2–11]. The complex and evolving nature of stigma presents a significant challenge in its measurement, as stigma can be experienced from many levels [12,13]. Trans-women face stigma on multiple levels (structural, interpersonal, and internalized) and their social identities place them in situations where they experience increased levels of marginalization and discrimination [2,14,15]. The experiences of stigma at all levels of society for trans-women have been primarily attributed to the social endorsement of cisgenderism (a system that delegitimized one’s ability to identify with a gender identity or expression different from society’s heteronormative beliefs) and anticipated stigma from observed and past experiences [2,16].

Within Sub-Saharan Africa, the stigma faced by trans-women is similar to that experienced in other parts of the world [17,18]. At the individual level, both enacted and observed stigma have led to an increased risk of depression and adverse mental health outcomes for trans-women [19,20]. Internalized stigma results in self-doubt and internal struggles, leading to poor adherence to medication and low interest in seeking healthcare, particularly in the field of HIV [21]. The environment in which trans-women live can significantly affect how they experience stigma [9,22]. In predominantly heteronormative communities, the likelihood of facing discrimination or marginalization increases due to society’s stereotyping of gender expressions and behaviors that are considered culturally unacceptable [3,4]. Recent studies have shown that stigma among trans-women often occurs within the family [23]. Although this phenomenon is not well studied in Ghana, limited research has indicated that people assigned male at birth who do not conform to social roles based on sex often avoid their families due to the stigma and discrimination they face from family members [4,7,24].

Residuals of colonist ideologist laws in Africa have also been streamlined to target nonheteronormative persons. Examples include the Same-Sex Marriage (Prohibition) Act of 2013 in Nigeria, the Penal Code of 1998 in Guinea, the past Criminal Code 250 of Mauritius, and proposed bills such as “The Promotion of Proper Human Sexual Rights and Ghanaian Family Values Bill” in Ghana [3,25,26]. The current LGBTQ+ bill (The Promotion of Proper Human Sexual Rights and Ghanaian Family Values Bill) in Ghana proposes imprisoning anyone whose gender identity or expression departs from socially accepted heteronormative beliefs [27]. The bill further criminalizes the production, marketing, dissemination, or association with LGBTQ+ groups or advocacy on their behalf [27]. Many studies have also highlighted a positive association between religious dominance and its influence on policies and legal strategies targeting nonheteronormative individuals [3,7,24,28]. These studies often point to more religious countries as having stricter laws against nonheteronormative individuals or those who support such ideas [3].

This study was conducted in urban slum communities due to the additional challenges these areas present, such as limited social infrastructure, poverty, unemployment and reduced access to social services [24,29–31]. For sexual and gender minorities, these challenges compound existing stigma from marginalization and discrimination [3,7]. Previous studies have shown that the stigma experienced by trans-women in low-income settings, such as urban slums, can lead to severe mental health outcomes, including suicidal thoughts, anxiety, and depression, which may result in behaviors like condomless sex [32–34]. While research shows increasing levels of stigma for trans-women in all facets of society, understanding how these stigmatizing experiences occur at various levels, including how trans-women internalize it, is imperative. This study is particularly crucial in underserved communities like urban slums to understand the unique needs of trans-women living there. This study highlights the unique experiences of stigma faced by trans-women in urban slum communities.

Beyond applying the socio-ecological framework descriptively, this study offers a theoretical contribution in three ways. First, it extends socio-ecological understandings of stigma by demonstrating how a highly salient punitive legal context (the proposed LGBTQ bill) functions as a macro-level driver that penetrates institutional, community, interpersonal, and individual domains for trans-women in urban slums. Second, the findings elaborate how ‘place’ (i.e., resource-limited urban slum settings) shapes the intensity and configuration of stigma mechanisms across ecological levels, thereby specifying context-sensitive pathways that have been under-theorized in prior work on trans stigma [12,35]. Third, by tracing connections between external structural forces and internalized self-doubt, regret, and social withdrawal, the study refines existing ecological stigma models to more explicitly link public policy and institutional practices to internalized stigma among trans-women in low-resource, criminalizing environments. Taken together, these insights move the ecological model beyond a purely descriptive categorization of stigma experiences, toward a theorization of how punitive legal regimes and place-based deprivation interact to generate specific cross-level pathways of transgender stigma in West African urban slums.

We employed an ecological model in this study. This model has been widely used in studying similar populations and has been instrumental in understanding and situating stigma experiences the framework [36–39], including among trans-women [12,35]. This model was used to guide our understanding of stigma among trans-women living in urban slum communities, and how these stigma experiences can be classified with the various levels of public policy, institutional, community, interpersonal, and individual/self-stigma.

## Methods

### Study setting

We conducted the study in two slum communities in the Greater Metropolitan Area in Ghana. Urban slum communities comprised of those located in Ashiaman, Mamprobi, Bukom, Teshie/Nungua, Labadi and their surroundings. These locations were purposively chosen due to their known description and characteristics as urban slums in Ghana [5,40,41].

### Research design

We used a phenomenological descriptive research approach [42,43] to investigate how trans-women in urban slums in the Greater Accra Metropolitan Area of Ghana experienced stigma related to their gender identity and sexuality. The phenomenological descriptive research approach seeks to understand how people experience phenomena in their daily lives, and captures the essence of these experiences from those affected [42,43]. This research approach was purposively employed in this study to capture the lived experiences of trans-women to share their stories and perspectives of stigma. Our goal was to identify the different types of stigmas experienced and categorize them based on the socio-ecological model.

### Sampling and recruitment procedures

In collaboration with a local community-based organization, individuals were recruited for the study using purposive and snowball sampling methods. These recruitment strategies, while appropriate for a hard-to-reach and criminalized population, likely favored trans-women who were already connected to community-based organizations or social networks, and may under-represent those who are more socially isolated, closeted, or disengaged from community structures. Recruitment was done with the help of research assistants from partnering community organization. We chose local community organizations as our partners because they have established connections with trans-women living in the urban slums.

### Inclusion criteria

To be eligible for this study, participants were required to meet the following criteria: (1) be 18 years and older, (2) live in an urban slum community in Accra, (3) self-identify as a trans-woman, and (4) have sexual intercourse with a man in the past year. Exclusion criteria included participants who were not assigned male at birth.

### Data collection procedures

Twenty participants were recruited through purposive and snowball sampling based on our prior research methods with this population [3,4,6–9,24]. In-depth, semi-structured interviews were conducted with trans-women in Accra, Ghana by trained research assistants. Before the interviews, eligible participants were given information sheets explaining the study and asked to sign consent forms. The research assistants clarified and reminded the participants about the important aspects of the consent documents during the data collection process. All interviews were carried out in a secure location provided by a community partner. Data for this study were collected from May 1^st^ to June 30^th^ 2023. All the in-depth interviews were conducted in English and audio-recorded using a digital voice recorder. The average length of time for interviews was approximately one hour. Stringent measures were implemented to ensure the protection of participants in this study. Trained research assistants with prior experience interviewing and working with gender-diverse individuals conducted the interviews. Furthermore, all interviews were held in discreet locations to guarantee participants’ safety. Participation was entirely voluntary, and all participants were thoroughly informed of their rights, including the freedom to participate or withdraw from the study at any time. Participants were also informed that in the event of adverse emotional and traumatizing feeling that may come up from the interviews, they can be provided information of organizations that specializes in trauma care, and will keep their identities discreet.

### Measures to prevent trauma among interviewers

To mitigate the risk of trauma among the research assistants conducting interviews, several safeguards were implemented. All interviewers received pre-study training on trauma-informed interviewing approaches, emotional boundary setting, and recognizing signs of distress in both parties (interviewer and interviewee). Interviewers were encouraged to take breaks between interviews to minimize emotional fatigue and were reminded of the importance of self-care practices throughout the data collection period. Additionally, referrals for mental health support were made available to interviewers should they experience emotional distress resulting from their involvement in the study.

### Interview guide

To ensure a comprehensive investigation of the research topic, an interview guide was created with the help of professionals in different fields, including trans-women living in urban slum communities, healthcare providers, and community organizations working with sexual and gender minorities. The interview guide contained open-ended questions designed to encourage people to share their personal experiences as trans-women, including gender and sexual discrimination that they faced while living in urban slum areas [4,6,7,44]. Questions asked during interviews comprised, among others, the following: [1] Does your family know about your gender identity? [2] What does your family think about you identifying as a trans-woman? [3] What has been your worst worry about being a trans-woman? [4] Can you recount a time you experienced stigma from the community? [5] Can you recount any time you experience stigma from the health facility?

### Analytical strategy

We coded and analyzed the qualitative data with the NVivo software (Version 14) utilizing content analysis [45,46]. Our team created a detailed guide for coding. The transcripts were coded, and similar codes were grouped to generate themes and sub-themes that accurately captured the experiences and perceptions of the participants. We did not calculate a formal kappa statistic; instead, we emphasized an interpretive consensus process. Disagreements were examined in team meetings, where coders explained their rationales, and the team reached consensus on code definitions and boundaries, leading to iterative refinement of the codebook.Initially, six research assistants worked together on the first five transcripts to reach a consensus on the coding process. The remaining transcripts were divided between two team members who independently coded them. This approach ensured consistency in our coding and allowed us to find common themes and subtle differences in the data. The team used coding comparisons and resolved discrepancies through group discussions to maintain consistency and accuracy in the coding process. The team also took diligent measures to remove any identifying information from the data, per ethical considerations. Finally, we identified key themes to represent the salient findings. During the transcription process, the team took care to remove any identifiers that could link responses to specific participants, ensuring their anonymity.

We monitored data sufficiency and saturation throughout data collection and analysis. After approximately 16 interviews, no substantively new codes or themes related to the multi-level stigma experiences of trans-women in urban slums were identified during team coding discussions. Four additional interviews were conducted to confirm the stability and depth of the emergent thematic structure. At 20 participants, we judged that thematic saturation had been achieved for the phenomena under investigation within this population and setting.

### Ethical approval and informed consent

This study was approved by the Research Subjects Review Board (RSRB) at the University of Rochester University (STUDY00008151) and the Ghana Health Service Ethics Committee (GHS-ERC:002/03/23).

### Reflexivity statement

The research team acknowledges that qualitative inquiry is shaped by the identities, values, and social positions of both interviewers and investigators. The team included Ghanaian researchers familiar with the sociocultural and political context surrounding gender diversity in Ghana, as well as researchers based in the United States with extensive experience working with marginalized communities, including gender-diverse populations. Some members of the research team have previous professional engagement with trans communities in urban slums, which supported rapport-building and sensitization to community priorities, while also requiring ongoing reflexive awareness to ensure interpretations were grounded in participants’ voices rather than researchers’ assumptions.

Throughout study design, data collection, and analysis, we engaged in reflective discussions to consider how our positionalities such as having academic privilege, varied gender identities, and differing degrees of proximity to the lived realities of trans-women in Ghana could influence interactions, analytic decisions, and representation of findings. We prioritized participant-centered interpretation by employing collaborative coding and consensus-building to reduce bias and ensure that themes authentically reflected participants’ experiences and perspectives.

Moreso, we recognize that our positions as academics, some affiliated with institutions in the Global North and some with longstanding relationships with local community organizations, shaped both access to participants and the framing of research questions. For example, our prior work on HIV and stigma in these communities sensitized us to legal and institutional drivers of stigma, which may have foregrounded these domains in our analysis relative to other concerns prioritized by participants. To mitigate these influences, we explicitly interrogated our assumptions during team meetings, invited community partners to critique our interpretations, and remained attentive to moments where participants’ accounts challenged our existing conceptual frameworks.

## Results

### Sample

The mean age of the sample was 20.0 (SD = 3.25, Range = 18–31). Fifty percent (n = 10) of the sample reported having tertiary education, 25% (n = 5) secondary education, and 20% (n = 4) primary education. One participant reported no formal education. Ethnically, participants of the Ga ethnicity were 40% (n = 8), Akan 30% (n = 6), Ashanti 20% (n = 4), and Ewe 10% (n = 2). Most of the sample identified as Christian (75.0%, n = 15). The average monthly income was GH₵830.15 ($70 USD). One hundred percent reported their relationship status as single. Fifty-five percent (n = 11) of the participants reported their type of housing as renting.

Our qualitative results revealed that at the public policy level, the LGBTQ+ bill (The Promotion of Proper Human Sexual Rights and Ghanaian Family Values Bill) in Ghana emerged as a significant driver of stigma. Subcategories associated with this bill included (a) restricted freedoms of expression and isolation from harm, (b) LGBTQ+ bill potential for facilitating violence, and (c) fear of arrest and jail time. At the institutional level, trans-women encountered stigma in three areas. The first was in the healthcare sector. The subcategory under this section was: (a) rude gestures and behaviors. The second institution where they experienced stigma was from law enforcement officers and subcategories under this included: (a) harassing attitudes from law officers, and (b) extortion. Community level stigma encompassed: (a) insults and name-calling, (b) possible harm from gender expression, and (c) fear of attacks from communities. At the interpersonal level, trans-women experienced: (a) invasion of privacy, (b) leaving home due to safety, (c) threat of bodily harm, and (d) rejection. At the individual/internalized stigma level, trans-women experienced: (a) self-doubt and internal struggles, and (b) sense of regret and guilt.

In reporting the findings, we prioritize an analytic account of how participants described stigma across ecological levels, using selected quotations to exemplify and nuance key patterns rather than to provide exhaustive narrative coverage.

#### Public policy level of stigma.

Participants revealed that the LGBTQ+ bill currently under consideration in Ghana’s parliament and awaiting presidential assent into law contributed to stigma for trans-women living in urban slums.

#### LGBTQ+ bill.

The presence of an LGBTQ+ bill created a sense of unease and concern among trans-women in the study. Within the LGBTQ+ bill were three subcategories: (1) restricted freedoms of expression and isolation, (2) LGBTQ+ bill potential for facilitating violence, and (3) fear of arrest and jail time.

#### Restricted freedoms of expression and isolation from harm.

Trans-women expressed fears that the passage of the bill could force them into hiding or expose them to the threat of imprisonment if discovered. According to some participants, the potential passage of the bill and its adverse repercussions could put them in jail for openly expressing as trans-women. Some participants indicated the inability to express themselves due to the potential harm that would come to them for identifying as trans-women affecting their ability to access healthcare services. Some trans-women indicated the bill had the potential to curtail freedom of speech and hinder their ability also to seek necessary support, such as counseling or therapy. These narratives from trans-women living in slum communities also revealed the deep fear, vulnerability, and marginalization they face, particularly in response to a potentially harmful bill. One woman expresses a fear of violence and social rejection, worried that the law will make it even more dangerous to exist in public, as she feels vulnerable to attacks without support. Another woman highlights the bill’s potential to erase her identity and suppress her ability to live authentically, pushing her further into secrecy and isolation. Both women also express concern about how these legal changes will impact their ability to earn a living, as the threat of increased discrimination could limit their economic opportunities and survival. Together, their stories underscore the emotional, psychological, and practical toll of living with fear of violence, legal persecution, and economic insecurity, highlighting the resilience required to navigate such oppressive systems. According to trans-women:

I thought about this bill. This bill [LAUGHS], okay, let me say that this bill they want to bring on board will not favor people like me. In other words, it is going to put me in the dark shadows. Things about me that need to come out for people to know who I am, are not going to come out because the moment I do, I may go to prison. - (23-year trans-woman)

In the long run, this bill that has been proposed to parliament is going to serve as a building to hide community members because as a community member, you cannot express your feelings, you cannot tell anyone what you are going through and even accessing health care puts you at the risk of going to prison. The bill prevents you from accessing healthcare at health facilities. - (26-year trans-woman)

I am scared because we are not welcomed in society, so when this bill is passed, maybe I can’t walk on the street anymore because I don’t know how I can manage it. I feel I cannot walk on the street because I feel like everybody can attack me at any time, anybody can attack me, and there might not be anybody to support me or to come to my aid, so, that is my biggest fear concerning this bill. If I can't even move out, how can I feed myself? How can I do something to earn something for my daily bread? so, it will really affect me. - (18-year trans-woman)

Let me say that this bill they want to bring on board will not favor people like me. In other words, it is going to put me in the dark shadows. Things about me that need to come out for people to know who I am are not going to come out because the moment you talk about it, prison. - (24-year trans-woman)

#### LGBTQ+ bill potential for facilitating violence.

Apprehensions concerning the passage of the LGBTQ+ bill into law extended to trans-women’s interactions with healthcare facilities, as they feared encountering stigma from healthcare professionals. Some trans-women mentioned the bill’s potential to incite violence against them, manifesting in various forms, including the destruction of their property, such as the burning of their shelter (Kiosks) by those opposed to LGBTQ practices and direct physical threats. Some trans-women indicated they felt the bill created an atmosphere of hate and resentment where they would get no help from anyone in the event they were being attacked in public. In one instance, trans-women shared that they deliberately avoided engaging in potentially confrontational situations. They described encounters where people around them made comments, such as expressing hopes for the government to expedite the LGBTQ+ bill so they could justify attacking trans-women in the community. Trans-women mentioned:

They say if the law is not passed, there will be no progress in the country. They will attack us; they will burn the kiosks that we sleep in so when I hear these things, I become very sad, so it will be better instead to remain the way we are now than to declare a passing of the bill. - (31-year trans-woman)

I feel like everybody can attack me at any time, anybody can attack me, and there might not be anybody to support me or to come to my aid, so, that is my biggest fear concerning this bill. - (25-year trans-woman)

This bill has made people violent to us. I remember one time I was walking, and then someone passed a comment, but I ignored it. The person said they should hurry and pass this bill quickly so they can deal with these people (trans-women). I heard it. But I ignored it. - (26-year trans-woman)

#### Fear of arrest and jail time.

Some trans-women indicated that the LGBTQ+ bill intensified their fears of being targeted by law enforcement officials, leading to their arrest and placement in rehabilitation facilities. Trans-women who engaged in sex work mentioned that the passage of the bill would create a situation where they could be targeted by law enforcement officers and arrested. Such a situation, according to trans-women, would create an environment where they felt threatened, leading them to stop working as sex workers. According to trans-women:

I think we should all be allowed to do the things we are comfortable with. The bill seeks to criminalize trans-women, and it bothers me. Now, I am not able to live in my community freely if the law is passed, I will be arrested and it worries me. - (22-year trans-woman)

The way I am, I am girly, so when the bill is passed, I might be jailed, so it is bad. - (19-year trans-woman)

I am a prostitute, and if they pass the bill and they see me, they will arrest me, so, how can I do my work. I am not working, so how can I eat if I dont do prostitution. – (30-year trans-woman)

The bill seeks to criminalize all MSM behaviors and for trans-women like me. This would mean that every LGBTQ member will either be jailed or sent to a rehabilitation facility. - (23-year trans-woman)

#### Institutional level stigma.

At the institutional level, trans-women experienced stigma in healthcare facilities and among law officers.

#### Health facility stigma.

Trans-women expressed their reluctance to visit healthcare facilities due to the perceived threat of encountering stigma from healthcare professionals. They described the hostile environment in the healthcare facility as dampening their enthusiasm for seeking essential healthcare services, such as HIV testing at these facilities. They also indicated the stigmatizing behaviors by healthcare professionals were dehumanizing. Under the health facility stigma was the subcategory, rude gestures and behaviors.

#### Rude gestures and behaviors.

Participants revealed a stark reality where trans-women faced significant barriers to accessing healthcare due to pervasive stigma and unwelcoming attitudes within the medical environment. Participant narratives highlighted a profound lack of protection and care by health professionals, leading to reluctance to seek medical assistance. Female nurses were singled out for their judgmental stares and rumored gossip, creating an environment where trans-women felt scrutinized for their gender expression. Trans-women mentioned this discomfort extended to crucial health services like HIV testing, where they described feeling dehumanized and marginalized by healthcare providers. Trans-women recounted:

There is no protection and care for trans-women. Many trans-women do not want to go to the hospital because of the stigma they experience. It is not just safe for them. Because if you go to the hospital, the female nurses are there. The way they stare at you. They won't say it, but when you go, they will say things behind your back. For me, they stare at me because I act like a lady. The way I walk and talk. That’s it. That’s why. - (26-year trans-woman)

I do not even feel encouraged to go to the hospital for HIV testing because of the stigma. You will see that the person has frowned his face and he’s sitting down there. I don't know. They'll frown their face and be sitting down there. After testing you, they will look at you awkwardly, as if you're not human. But you're working humans so you have to get some tolerance to human beings. - (27-year trans-woman)

#### Law enforcement stigma.

Trans-women described distressing encounters with law enforcement officers, where they were subjected to harassment and discriminatory treatment based on their gender expression. Their accounts revealed instances of unwarranted questioning regarding their appearance and behavior, with police officers questioning why they present as feminine. Moreover, trans-women reported heightened vulnerability to police scrutiny, as mere suspicion of cross-dressing could escalate into targeted investigations by law officials, perpetuating a cycle of surveillance and intimidation that further marginalizes the community. Within Law Enforcement Stigma, two subcategories emerged: [1] harassing attitudes from law officers and [2] extortion**.**

#### Harassing attitudes from law officers.

Trans-women shared that they experienced constant anxiety regarding law enforcement officials, who frequently engaged in stigmatizing behaviors, such as harassment, based on their gender expressions. Merely walking or dressing in a manner consistent with their gender identity or possessing feminine physical features was enough to attract unwanted attention and frequent interrogation from the police. According to trans-women:

Recently, I got stopped by the police, and they were like, why do I look like a woman, and I'm not even in cross-dressing attire, and they just ask me, why do I look like a woman? Why do you behave like a girl? why do you walk like a girl? These are some of the things I face. - (24-years trans-woman)

You will be there and before you realize the police will be coming for you, because once the police have heard that someone has cross-dressed, they will add things to the report to make it investigated so that the police will come after you. - (20-year trans-woman)

#### Extortion.

Trans-women recounted instances where law enforcement officers extorted them after being exposed as individuals engaged in same-sex activities. They described situations where they were arbitrarily arrested for engaging in non-heterosexual behaviors and coerced into paying exorbitant sums of money for their release. According to trans-women:

My friend, who is also a trans-woman, had a physical encounter with a man. After their encounter, this man called the police on my friend. When the police arrived, I was with my friend, and they tried to arrest both of us. At that moment, members of our community intervened, saying I was not a trans-woman. However, my friend insisted that I was, claiming that I also engaged in sexual activity with men. We were both arrested and taken to the police station. The police demanded a payment of 2500 cedis, but we explained that we didn't have the money. They reduced the amount to 1500 cedis. We still couldn't pay, so they insisted we pay 300 cedis each, threatening to keep us detained if we didn't comply. - (18-year trans-woman)

#### .....Community-level stigma.

Stigma experiences for trans-women within the community manifested in several ways, including [1] insults and name-calling, [2] possible harm from gender expression, and [3] fear from attacks.

#### Insults and name-calling.

Trans-women reported being called “Kojo Besia,” a local slang term in the “Twi” language spoken in southern and central Ghana denoting a man perceived to exhibit feminine traits, or derogatorily as “Batty boy.” To avoid the hurtful effects of this degrading name-calling, trans-women explained that they often chose to ignore individuals within their communities who engaged in anti-trans behavior, and preferred to avoid any potential confrontations that might escalate into such encounters. Trans-women recounted:

Okay, so for me right now, my decision is not to pay attention to negative vibes or homophobes so I can be walking. Yes. Sometimes I hear them call me *Kojo Besia* (man-woman). I'll just ignore like I didn't hear anything, and then move my way. If I sense harm coming, I will probably get a taxi or an Uber to jump in and go. - (22-year trans-woman)

They were like oh “Kojo Besia,” you are gay, you are so this and that, and me too I don't care and they also know that I don't care, I'm even tired of their name-calling. I don't use their words for anything. - (31-year trans-woman)

I face a lot of community stigma. Yes, a lot. While growing up, I had these features, big ass, and my gestures, they were like. How can a man behave like this? That kind of tag, you know, being called me Kojo Besia (man-woman), and a whole lot of names - (26-year trans-woman)

Ahhh well [LAUGHS] hmm, at some point in time, some people can see you and just insult you for no reason. Some will make a mockery out of you. Sometimes, even when braiding your hair and coming to pass, they will insult you. - (20-year trans-woman)

I was with my friends and from the way they were dressed, it was easy to identify us, so when we were passing by, the guys were insulting us, saying things like hey gay, batty boys, and names like that. - (27-year trans-woman)

#### Possible harm from gender expression.

Participants shed light on the intense pressure trans-women in slum communities face to conceal their gender identity in public spaces due to the risk of violence, legal repercussions, and social rejection. A trans-women explained how she felt forced to cross-dress only in private, as going out in feminine attire would expose her to potential arrest by the police and violent retribution from the community. Another participant elaborates on the need for constant vigilance, noting that trans-women must carefully navigate when and where to express femininity to avoid physical attacks or harassment, even in places like hospitals. Both participants described the coping mechanism of dressing in men’s clothing as a survival strategy, masking their true identities to avoid danger. Tran-women mentioned:

I cross-dress only when I am in the room and when I am with my guys. You cannot cross-dress outside. In as much as you can wear certain feminine things out and it is nobody’s business, cross-dressing entirely with make-up and wig on and you go out, if you don't take care the Inspector General of Police will come and arrest you. The community themselves will kill you. I haven't had any personal negative experiences like that, but the kind of comments that come at you, you're not encouraged to cross-dress. You are forced to be in men’s clothing, hide yourself, and pretend you are not trans. Dressing as a man is a coping mechanism. - (30-year trans-woman)

You see, in Ghana, there are actually too many attacks. So you have to know where and when to act feminine. Because, in the sense that if you're in your home and want to go out in feminine clothes, you might not know who is following you. You don't. if you meet a homophobic on your way to the hospital, you might just get attacked because of how you are dressed; you just have to put yourself first and then just be in the dress the community expects you, that is the sex assigned to you at birth. - (24-year trans-woman)

#### Fear from attacks.

Despite efforts to navigate through unsafe areas and exercise caution, the threat of violence from the community remained ever-present, instilling a deep-seated fear of harm and even death. Given these potential threats, trans-women mentioned it was important to constantly remind themselves to stay safe from harm. According to trans-women:

My worst fear is being beaten or attacked by homophobes because each day, they will be like, you have to take care of yourself. Be careful. And then these areas don't go there. I heard this story, so just be careful. They keep reminding me of my safety. - (19-year trans-woman)

It’s not everybody that likes us. Some may like you, some won't like you. So I'm scared. They can just kill you and then dump you somewhere. That’s my greatest fear that someone might do something to me. So when I'm working I'm very steady. - (20-year trans-woman)

#### Interpersonal level stigma.

Trans-women described their experiences of interpersonal stigma to exist between themselves and members of their family. Stigma under interpersonal level stigma were subcategories: [1] invasion of privacy, [2] leaving home due to safety, [3] threat of bodily harm, and [5] rejection.

#### Invasion of privacy.

Family stigma significantly impacted trans-women, who narrated facing discrimination from their relatives due to their gender expressions. According to trans-women identifying as trans often led to their private matters, which ideally should not concern their families, being brought up in family gatherings. Participants explained these meetings were orchestrated to shame and humiliate them for their involvement in non-heterosexual or gender non-conforming activities. Trans-women also shared instances where their family members went as far as invading their privacy by accessing their phones to share personal messages and photos with other family members, all with the intention of publicly humiliating them and inciting confrontation. According to trans-women:

Okay, so it wasn't easy for me. From the onset, I didn't really come out to them, I was exposed. So it wasn't that easy. Because I was being called in front of the family, the extended family, and then being accused of sleeping with a fellow man was very, very not easy for me. - (19-year trans-woman)

A cousin visiting from Nigeria borrowed my phone after losing his. But I started realizing he was looking at my messages, so I changed the password to prevent further access. However, he manipulated a kid in our house who knew the password to retrieve my new password and shared screenshots of my private chats with the family. I was confronted by my mother, aunts, and sisters. I felt so helpless and unable to defend myself. I just stood there in tears in front of the family. - (20-year trans-woman)

#### Leaving home due to safety.

Trans-women mentioned they felt compelled to flee from their families due to the threats of physical violence. For them, seeking refuge elsewhere meant finding a safe environment away from relatives who endangered their well-being. They expressed feeling abandoned by other family members who failed to support them when their families accused them as one of the reasons why they decided to move away from their families to different locations. One trans woman shared how her brother threatened her, forcing her to flee for safety, and highlights the emotional distance and estrangement from her family, who are unaware of her exact whereabouts. This story reflects how familial rejection can lead to a loss of safety, security, and belonging, pushing trans-women to leave their homes to survive. Another trans woman described a similar experience, where her family’s hostility and insults led her to distance herself from them, avoiding further emotional harm. Trans-women recounted:

I used to be in Sunyani, so I was threatened by my own brother that he was going to torment my life. He’s really going to make life hell for me. So I had to run cause I felt I had to go cause I was not safe at that place. So it’s like there’s a big, um, How should I put it? There’s a big gap between me and my family, so nobody’s really supportive. They don't even know my whereabouts; they know I'm in Accra but don't know where I am staying. Yeah. - (31-year trans-woman).

Unfortunately, when they found out I wasn't currently in the house, I moved out before they found out. After a couple of years, I went back to the house, and they started like, they were not okay with me from the beginning at all, sometimes kind of insulting me by saying all kinds of things and stuff. From then, I stopped getting closer to them, I gave myself some distance between me and my family, so, I did not experience much hatred from them, because when they started behaving badly towards me, I decided to cut them off for some time. - (24-year trans-woman).

#### Threat of bodily harm.

Trans-women recounted disturbing experiences of family members threatening them with physical harm due to their gender and sexual preferences. These threats stemmed primarily from their families’ disapproval of such gender identities and behaviors, leading them to resort to violence. Trans-women noted that these threats mainly came from family relatives who did not live in close proximity to them, who later came to live around them and discovered their identities. According to trans-women:

I remember my grand mom’s funeral, this community came around and one of my uncles from Togo came and was like, why is the family condoning such act and they are allowing me to have this kind of freedom? if so, they have to catch me and beat me. My mom has to deal with me and handle that situation. They had to call a general family meeting where I was and I had to account to them all. - (24-year trans-woman)

There was a time my own brother threatened to raise hell on me for being a trans-woman. He even threatened to beat me up. I did not feel safe around so I moved to Accra (city). So it’s a big problem in my family. – (20-year trans-woman)

#### Rejection.

Trans-women highlighted that the stigmatizing behavior of their families made them feel rejected. They recounted how their gender and sexual identities caused their families to feel ashamed of their participation in family activities and gatherings. Participants also mentioned that being trans-woman meant their opinions and contributions to family affairs were not valued or considered worthy. Even in instances where they attempted to establish a relationship with their family, trans-women still receive rejection from the family. Trans-women mentioned:

Oh, usually when there is a family gathering, and no one wants you to be there because when you appear, they'll start asking whose son that is. It goes down to trace to the family. And to them, they feel like you are tarnishing the image of the family. You are giving the family a bad name. So that’s one of the stigmas, and aside from that, how they speak to you, or certain issues you want to contribute but since you're a trans-woman, they feel like there is no use of your opinion or something. - (18-year trans-woman)

Unfortunately, when my family found out about my trans status, I wasn't currently living in the house, I moved out before they found out. After a couple of years, I went back to the house, and they started like, they were not okay with me return back to the house. Sometimes they will insult me by saying all kinds of things. From then, I stopped getting closer to them, I gave myself some distance between me and my family because of the rejection. – (19-year trans-woman)

#### Individual/Internalized-stigma.

Accounts of internalization of negative stereotypes among trans-women showed varied accounts of stigma where they began to internalize the negative attitudes from society. Within the interpersonal level of stigma were the subcategories: (1) self-doubt and internal struggles and (2) sense of regret and guilt.

Trans-women mentioned the challenges of dealing with inner conflicts rooted in thoughts about their gender identities and questioning whether identifying as a trans-woman as inherently wrong or similar to being labeled a demon. Some trans-women expressed discomfort in their roles, particularly when working as food vendors for individuals who may not accept their gender identities. This discomfort was compounded for those who also tested positive for HIV, as they felt it was unfair to serve food to people who rejected their gender identity. According to trans-women:

#### Self-doubt and internal struggles.

Sometimes I ask myself, is it true that we were created like this? Are we demonic or something? Sometimes I do question myself, I'm trying to find out the answers to these kinds of questions. - (23-year trans-woman)

Yeah, I was discriminating myself, self-stigmatizing, because I'm into food, so, I was like, how can I cook for people again. So, mentally, I started questioning myself, me that have the HIV infection preparing food for people to eat. I get scared. (26-year trans-woman)

#### Sense of regret and guilt.

Some trans-women also mentioned regret and guilt regarding their trans identity. They indicated that being trans had hindered their ability to achieve significant life goals, including educational support from others who would have helped them if they did not identify as trans-women. Additionally, some participants blamed themselves for being physically maltreated by their communities, attributing the attacks to their choice to live openly as trans-women. These thoughts caused a profound sense of shame among some participants. Trans-women mentioned:

My worst worry is to, um, regret being like this, being a trans-woman. As in, um, now I don't have access to education again. I can't further my education, and I can't really have a better life compared to. Previously being the male I used to be, I would, I would've been, um, sponsored to school. Someone would take care of me and all of that. So me being a trans and not enjoying such privileges, I am scared. Cause I'm like, where is this all going to? Where will it end? And yeah, that is the case with me. - (20-year trans-woman)

I will stay all day indoors. I will be thinking a lot and asking why God created me like this because I even wonder why people treat me like that, throwing stones at me and threatening me. Sometimes, I even feel ashamed to go outside. - (30-year trans-woman)

## Discussion

The experiences of stigma can occur on many fronts across various levels of society [24,47–49]. For trans-women, these stigma experiences have been observed to occur at the highest levels of public policy down to the point where they internalize such stigma [50–52]. Additionally, places where individuals live have been a significant determinant of stigma for individuals within the LGBTQ+ community [4,53]. Trans-women living in resource-limited communities, such as urban slums, face additional stigmatizing challenges [8,54]. In this study, we adopted a socio-ecological framework of stigma [55] for trans-women living in urban slum communities to describe the various types of stigmas that occur within the public policy level of stigma, institutional level stigma, community-level stigma, individual/internalized-stigma, and interpersonal level stigma. Our findings build on prior ecological stigma scholarship by theorizing how punitive legal environments and the constraints of urban slum settings jointly structure multi-level stigma pathways for trans-women, thereby extending the socio-ecological model to contexts where criminalization and extreme resource scarcity are central macro-level conditions.

In many sub-Saharan African countries such as Ghana, such legal protections are not in place to ensure the safety of trans-women [56,57]. Current legislation in various African countries, including Nigeria’s Same-Sex Marriage (Prohibition) Act of 2013, the Criminal Code of Ethiopia, Proclamation No. 414/2004 of Ethiopia, Guinea’s 1998 Penal Code, Malawi’s Penal Code Chap. 7:01, and Mauritius’ former Criminal Code 250 (which was later invalidated by the country’s Supreme Court), all impose penalties on individuals who do not conform to traditional gender norms. These laws act as legal tools to suppress same-sex activities and relationships [3,26]. These legislations directly contrast the principles outlined in the Universal Declaration of Human Rights, which advocates for the protection of individual rights and freedoms, regardless of gender or sexual orientation. The Universal Declaration of Human Rights adopted by the United Nations in 1948, asserts that all people are entitled to equality, dignity, and the right to freely express themselves without discrimination.

Currently in Ghana, laws being proposed in the Ghanaian parliament such as the “The Promotion of Proper Human Sexual Rights and Ghanaian Family Values Bill,” propose to criminalize same-sex intercourse, gender-non-conforming behavior, same-sex marriage, or any form of advocacy for LGBTQ + , with victims facing up to five years in prison for any or related offences [58,59]. Our findings among trans-women living in urban slum communities revealed the proposed LGBTQ+ bill in Ghana created a sense of fear of freedom of expression. This sense of fear has been observed with similar bills within Sub-Saharan Africa [3,25,26,60–64]. Trans-women often had to hide or avoid encounters to conceal their identities and avoid being recognized in society by those who did not accept their gender identities and behaviors [60,61,65]. Our study also observed instances where the potential passage of this legislation could lead to jail time for trans-women. This fear reflects specific clauses in the Ghanaian LGBTQ+ bill that enforce criminal sanctions on any activity deemed to undermine Ghanaian family values and proper sexual human rights, as outlined in the bill [59]. Also observed in many parts of Africa, laws and bills that suggest the curtailing of rights and freedom of people in the LGBTQ+ community have resulted in the avoidance of proper health access and services for members of these communities [66]. Laws that criminalize the identity and activities of people in LGBTQ+ have direct and indirect consequences on how they access health care or experience services that are rendered [67,68].

Stigma, particularly for trans-women, can have significant health consequences related to healthcare utilization and service provision [60,61,69]. Studies in Africa have shown multiple forms of stigma against non-heterosexual, and gender diverse communities [11,70–72]. Within the domain of HIV prevention and care, LGBTQ+ individuals have described their experiences at health facilities as being marked by neglect and disregard by health professionals [8,73,74]. Our study participants’ experiences of being treated rudely by health professionals in urban slums mirrored similar experiences of trans-women and other sexual minority groups in different parts of Africa [3,24,54]. Given the hostile climate in Ghana regarding the acceptance and recognition of LGBTQ+ individuals, earlier studies have suggested that this atmosphere can foster hate and bias among health professionals when providing treatment, especially in urban slum communities [4–6,8]. Our findings indicate that this climate of hate still exists and is common among health professionals in Ghana.

Historically, sexual and gender minorities have expressed concerns about targeted activities that seek to criminalize their gender identities and behaviors [3,25,26,59]. Studies have shown that trans and other gender non-conforming individuals have some of the highest incarceration rates, with Black trans-women being disproportionately affected [75,76]. In Ghana, the existing insecurities among LGBTQ+ individuals has been propagated from the proposed Proper Sexual Rights and Ghanaian Family Values bill, which contributes to the adverse treatment of people who identify with these communities [3,9]. Findings from this study suggest that trans-women continued fear of targeted criminalization and mistreatment, particularly from law officials. Many participants expressed concerns about harassment from law officials, particularly around unlawful arrests, extortion, and detention in prison simply because they identified as trans-women.

Within the sub-Saharan region of Africa, many communities have proven unsafe for individuals who identify with or sympathize with the LGBTQ+ community [5,9,24,77]. Trans-women have been subjected to various forms of microaggressions and dehumanizing conditions, ranging from insults to name-calling [78–80]. Our findings confirmed these findings from earlier research, with participants indicating that they faced multiple forms of discrimination, including name-calling and insults from people who did not accept their gender identity or behaviors within the urban slum community.

For trans-women, gender expressions such as cross-dressing could be a means of expressing their gender identity rather than their sexual orientation [81,82]. Historically, cross-dressing has been associated with cultural and traditional practices and has not necessarily attracted stereotypical behaviors from individuals with heterosexual beliefs [81]. However, recent studies have demonstrated a direct correlation between one’s gender identity and the likelihood of facing discrimination and marginalization in their communities when they cross-dress [81]. Moreover, the level of social acceptance of cross-dressing among LGBTQ+ individuals vary significantly across different communities [27,83,84]. While there is a general level of acceptance for trans-women in many Western countries [84], the same cannot be said for many African countries [3,9,25,26,62–64]. Reports of physical attacks and legal policies targeting the freedoms of LGBTQ+ individuals who cross-dress have been documented in several African countries [9]. Our study confirms these assertions in Africa, indicating that trans-women face significant threats when cross-dressing [83]. In many instances, they must conform to the community’s traditional male and female clothing standards to ensure their safety [3,81].

Our findings revealed that the invasion of privacy by family members emerged as a form of interpersonal stigma experienced by trans-women living in urban slum communities. Current research in Ghana has documented adverse family reactions to the gender identities and expressions of sexual minorities in these areas [7]. Studies have also shown that the control and surveillance by family members can significantly impact the lives of sexual and gender minorities, particularly when these intrusions reinforce heteronormative standards [7,85,86]. In this study, trans-women reported similar challenges when their privacy was violated or when they were pressured to conform to a heteronormative lifestyle. This violation of privacy not only fosters a sense of helplessness among trans-women but also increases their vulnerability to stigma [7].

Participants in the study expressed the need to evade the invasion of privacy and the push to live a heteronormative lifestyle from family members by running away from spaces where these sentiments were pushed. Although there is a void in research on trans-women in Ghana, studies on non-heterosexual, gender-diverse people and individuals who identify with the LGBTQ+ community show the forced displacement of these individuals in urban communities [7], due to family rejection and violence [87,88]. Non-heterosexual, gender-diverse people and individuals who identify with the LGBTQ+ community have been compelled to seek refuge in places where they have felt safe, away from family leading to social isolation and marginalization [7]. The accounts of trans-women relocating to different cities in this study for safety underscore the severe impact of familial stigma on their lives, and the continuous need to evade the invasion of privacy from their family members.

Participants also stressed the recurring verbal threats they received from their family members. Such threats were mainly attributed to disapproval of gender identity or behaviors reflecting a broader societal pattern of violence against LGBTQ+ individuals. While the family has historically been studied as a source of support for individuals [89], the same cannot be said for LGBTQ+ individuals [7,90]. Recent studies in Ghana have shown the hostile climate for LGBTQ+ individuals that extends beyond the family level, with reports of stigma coming from close family members [3,7]. Our study reinforces these recent findings among LGBTQ+ individuals living in slum communities and shows family rejection of their gender identities and expressions persists. Trans-women described how their families’ stigmatizing behaviors made them feel unwelcome and unvalued, particularly during family gatherings. This form of stigma is well-documented in the literature as a critical factor contributing to the social and emotional isolation of individuals who belong to the LGBTQ+ community. The participants’ experiences of being excluded from family activities and having their opinions disregarded further demonstrate the pervasive nature of familial stigma and its detrimental impact on their sense of belonging and self-worth.Top of Form

Further, our findings showed that trans-women’s experiences of self-doubt and internal struggles were the result of internalized stigma. Such experiences of internalized stigma have been indicated in previous research and shown to reflect the internalization of negative societal behaviors towards diverging gender identity and behaviors of sexual minorities. Participants’ indication of internalized stigma was related to the profound inner conflict linked to their gender identity, questioning if their existence as trans-women was inherently wrong or demonic. This self-doubt and psychological state of thinking aligns with the concept of “identity incongruence,” where the internalization of societal rejection leads individuals to doubt the legitimacy of their identities [91]. Trans-women also indicated living with HIV heightened their levels of self-doubt and internal struggles. These experiences align with findings from other studies on trans-women living with HIV where self-stigma could result in no or poor HIV medication adherence [92]. The sense of regret and guilt reported by trans-women also reflects the effects of social rejection on self-perception and life opportunities. Trans-women in this study regretted the loss of educational and professional opportunities, attributing these losses to their gender identity.

This sense of regret is reflective of the broader societal barriers through the marginalization and discrimination that trans individuals face, which often lead to limited access to education and employment [93–96]. More so, trans-woman’s feelings of guilt and self-blame as indicated in the study align with the concept of “secondary victimizations” [97,98], a term that seemingly suggest circumstances where trans-women experiences of stigma from marginalization and discrimination they receive from society leads to self-blame and psychological stress. The social withdrawal of trans-women indicated in the study are consistent with the effect of secondary victimization and the effect of internalized stigma on social functioning.

Our use of the ecological model was aimed at providing a better understanding of the multifaceted experiences of stigma among trans-women living in urban slum communities in Ghana. Conceptually, these results suggest that existing socio-ecological models of stigma should more clearly articulate [1] the role of specific legal instruments as proximal policy mechanisms, and [2] the mediating function of place-based deprivation in translating policy-level stigma into community, interpersonal, and internalized stigma for trans-women. Thus, our study contributes theoretically by specifying (a) policy–place–stigma linkages that are largely absent in existing ecological stigma models, and (b) mechanisms through which these macro-level conditions cascade into interpersonal and internalized stigma among trans-women in criminalizing, resource-limited settings. By examining stigma at various societal levels, including public policy, institutional, community, interpersonal, and individual/self-stigma, the model offered a comprehensive analysis of how these different levels interact and compound the stigma experienced by individuals. The model has been previously used to guide stigma studies among trans-women [12,35]. It also highlights key drivers of stigma in this study, such as the proposed LGBTQ+ bill in Ghana at the policy level and stigmatizing behaviors in healthcare and law enforcement at the institutional level. The model also emphasizes the role of place, showing that trans-women in resourced-limited areas face unique challenges. Our previous research on trans-women and similar populations have revealed the nuanced experiences of stigma for these communities [3–9,24,40]. Additionally, the model underscores the lack of legal protection for trans-women in many sub-Saharan African countries [3,59,92], drawing attention to the urgent need for policy changes. Overall, the socio-ecological model’s holistic approach informs the development of multi-level interventions and advocacy strategies to reduce stigma and improve the well-being of trans-women.

## Limitations

One notable limitation in this study involves generalizing the experiences of trans-women regarding stigma in Ghana. Moreover, as a qualitative study with 20 participants, our findings are not statistically generalizable. However, within this sample and setting, we observed thematic saturation of the main stigma processes across ecological levels, suggesting that the data were sufficient to support the analytic claims advanced here. Second, our use of purposive and snowball sampling through community-based organizations may have introduced sampling bias toward trans-women with stronger community ties or service engagement. Trans-women who are not connected to such organizations, including those who are more hidden or precariously housed, might experience stigma differently than those represented in this sample. Third, our inclusion criterion requiring sexual intercourse with a man in the past year further narrows the sample and limits the transferability of findings to trans-women whose recent sexual histories differ. Given the high number of urban slum communities in Ghana, a more comprehensive study capturing the experiences of trans-women across geographical regions in Ghana would provide a better reflection of their experiences of stigma, taking into account factoring in other key variables such as religion. Ghana is predominantly Christian in the southern regions and predominantly Muslim in the Northern Regions [99]. Our findings are based solely on samples from trans-women in Ghana’s southern region, without including samples from the country’s northern region. Consequently, we are unaware of the role religion may play in the intensity or nature of stigma among trans-women living in other parts of Ghana. Expanding the study to include these areas could provide a more comprehensive understanding of the factors influencing stigma across different regions and communities.

## Conclusions

Tackling the unique needs of trans-women, particularly those living in urban slum communities, requires multi-level responses aligned with the socio-ecological nature of the stigma processes we identified. At the public policy level, participants’ fears surrounding the proposed LGBTQ bill and potential incarceration underscore the importance of legal and policy reforms that decriminalize gender diversity and protect gender-diverse people’s rights, which prior work has linked to improved health and safety outcomes for LGBTQ communities in other settings. The public policy level points to concrete legislative priorities for Ghana and similar contexts. In addition to opposing the passage of the Promotion of Proper Human Sexual Rights and Ghanaian Family Values Bill and related punitive laws, policymakers should (a) enact anti-discrimination protections explicitly covering gender identity and expression; (b) remove legal and regulatory barriers that discourage trans-women from seeking healthcare and social services; and (c) incorporate protections for gender-diverse people into national health, housing, and violence-prevention strategies, in line with international human rights guidance and emerging evidence on the health benefits of decriminalization and protective legal frameworks for LGBTQ communities.

At the institutional level, narratives of rude treatment by healthcare professionals and harassment and extortion by law enforcement point to the need for structural interventions, including provider training, accountability mechanisms, and protective guidelines that have shown promise in reducing stigma in health and justice systems. At this level findings support the development of binding standards and accountability mechanisms within health and law enforcement systems. For healthcare, these include mandatory pre-service and in-service training on transgender-affirming care, confidentiality protections for gender identity disclosure, and stigma-reduction programs integrated into facility quality-improvement initiatives. For law enforcement, policy responses should establish clear prohibitions and sanctions for harassment and extortion based on gender expression, alongside community monitoring mechanisms and training on human rights and gender diversity for police officers.

At the community and interpersonal levels, experiences of name-calling, threats, and familial rejection suggest that community-based stigma-reduction and family-focused interventions may be critical for mitigating violence and social exclusion, consistent with emerging evidence on family-based and community stigma interventions with LGBTQ populations. At the individual level, participants’ accounts of self-doubt, regret, and social withdrawal highlight the need for trauma-informed, affirming mental health services that directly address internalized stigma and secondary victimization, building on prior work on internalized stigma and its mental health consequences among sexual and gender minorities. At the community and interpersonal levels, policies could support community-led safe spaces, peer educator programs, and family-based education initiatives that address misinformation and promote acceptance of gender diversity. At the individual level, integrating trauma-informed, trans-affirming mental health services into existing HIV and primary care platforms may offer a feasible route to expanding access in resource-constrained slum settings

Our findings, grounded in a socio-ecological framework, provide a context-specific yet transferable account of how multi-level stigma operates for trans-women in urban slum settings in Ghana. By explicating pathways from policy and institutional contexts to community, interpersonal, and internalized stigma, this study offers a theoretical and empirical foundation for designing multi-level interventions to reduce stigma and improve the quality of life for trans-women and other gender-diverse people living in similar criminalizing, resource-limited environments.
